# Arteriovenous fistula after robotic partial nephrectomy: Case report and narrative review^☆^^☆☆^

**DOI:** 10.1016/j.radcr.2022.04.038

**Published:** 2022-05-17

**Authors:** Antonio Tufano, Vincenzo Asero, Flavia Proietti, Rocco Simone Flammia, Giorgio Franco, Costantino Leonardo

**Affiliations:** Department of Urology, Sapienza University, Policlinico Umberto I Hospital, Viale del Policlinico 155, 00161, Rome, Italy

**Keywords:** Arterio-venous fistula, Vascular lesion, Partial nephrectomy, Embolization, AVF, Arteriovenous fistula, PN, Partial nephrectomy, MIPA, Minimally invasive partial nephrectomy, eGFR, estimated glomerular filtration rate, SA, Selective angiography, SAE, Selective angiography embolization

## Abstract

Arteriovenous fistulas (AVF) of the kidney are uncommon. They may be acquired, idiopathic or arise of congenital arteriovenous malformation. Acquired renal AVF are mostly iatrogenic due to the increasing number of mini-invasive nephron surgery. We report a case of renal AVF in a 65-year-old woman previously treated with left robotic partial nephrectomy (PN), which was successfully treated by endovascular coiling.

Arteriovenous fistulas (AVF) of the renal artery branches are uncommon complications of partial nephrectomy (PN). They usually occur after invasive procedures, such as percutaneous nephrostomy, renal biopsy, penetrating trauma and surgery. There are a limited number of reports describing the presentation and management of this rare complication. Incidence of AVF is reported to be of 1% after open approach and 1.96% after minimally invasive approach [1].

However, AVF can potentially represent a serious complication of PN and typically requires selective angioembolization to control the bleeding.

We herein report a case of late AVF after minimally invasive partial nephrectomy (MIPN) and reviewed the literature in order to provide useful information to improve the awareness of AVF among clinicians, reduce missed diagnoses, and achieve an accurate diagnosis and treatment.

## Case presentation

**Fig. 1:**
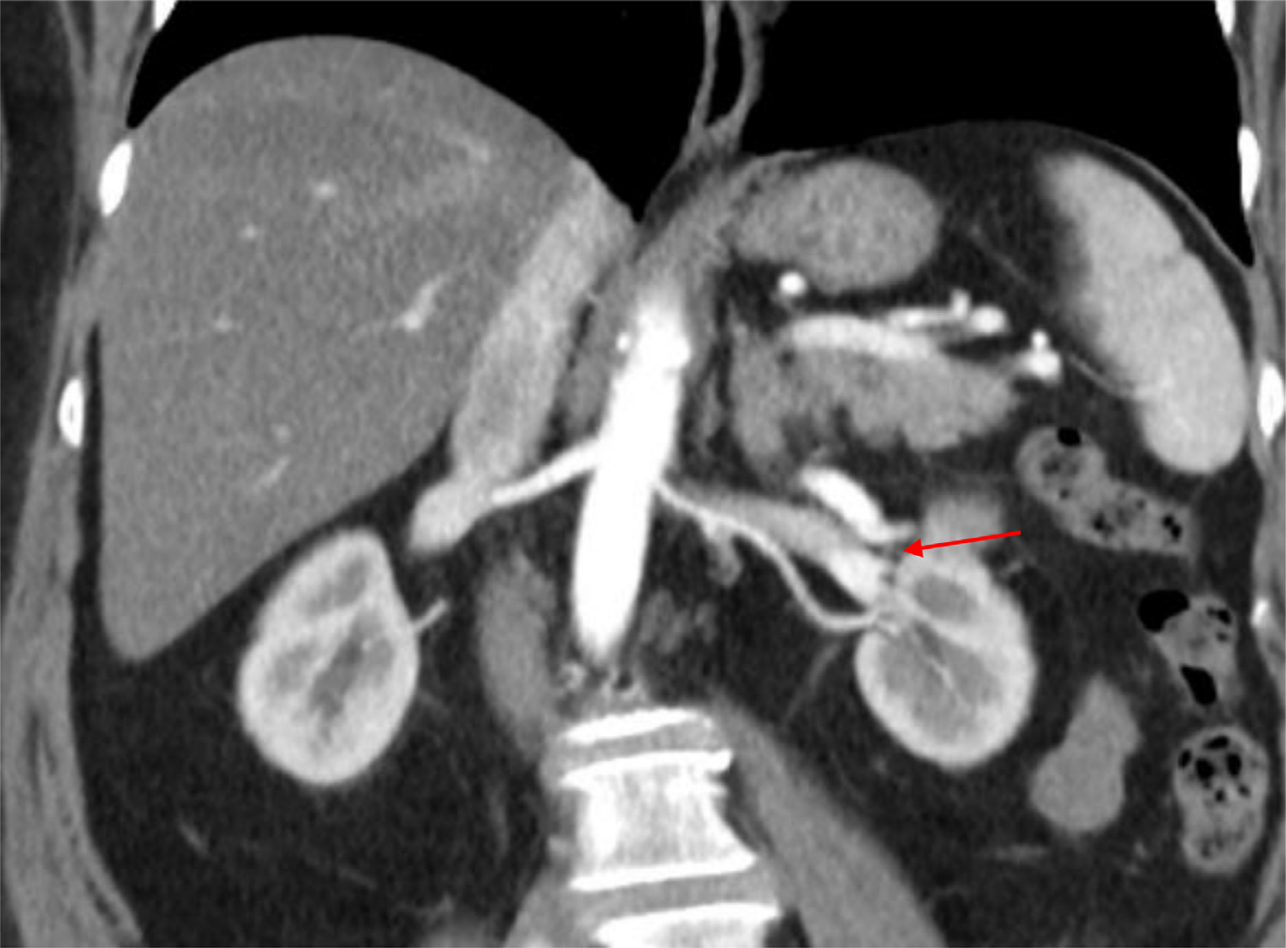
CT arterial phase angiogram shows left AVF (arrow)

**Fig. 2:**
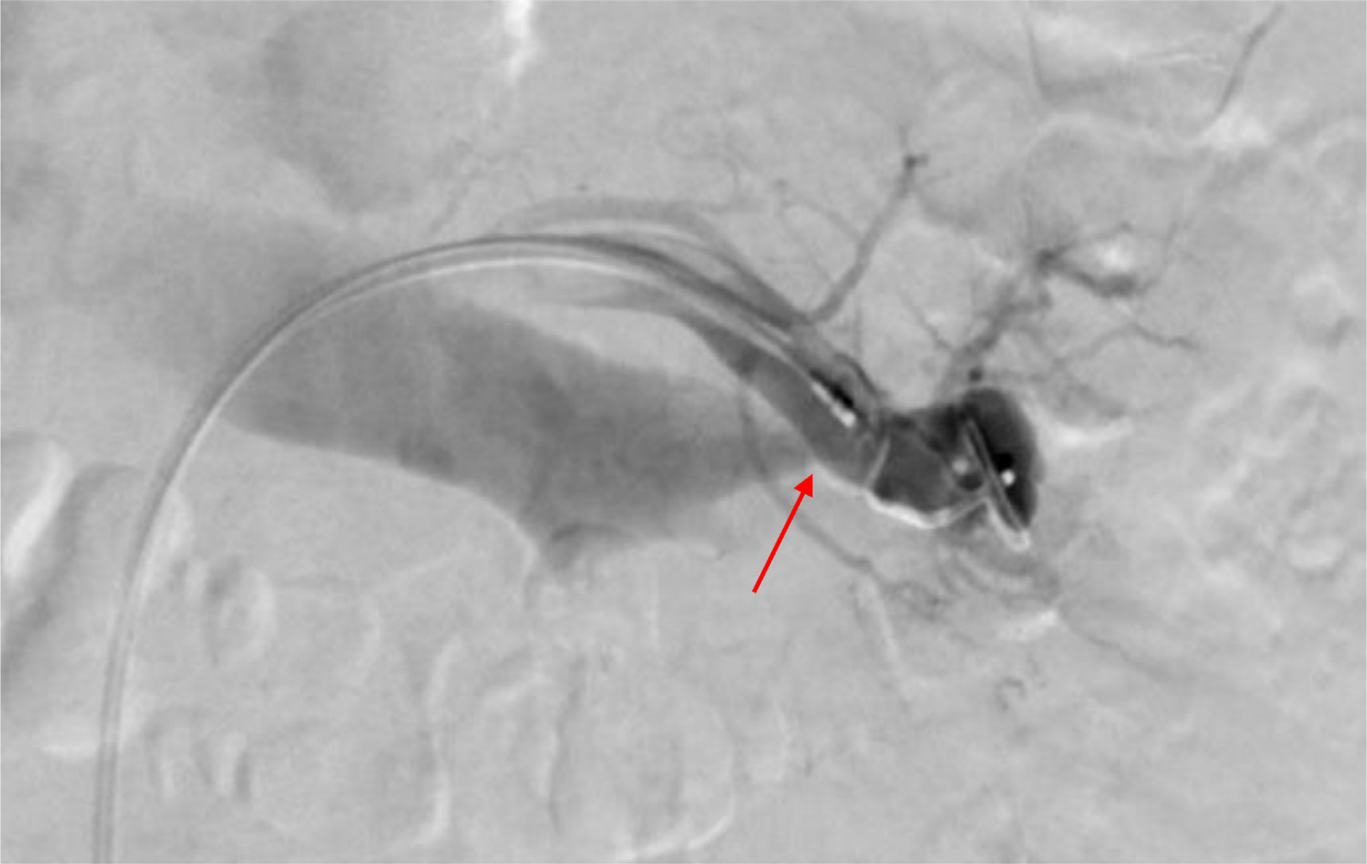
Angiographic image of left AVF (arrow)

**Fig. 3:**
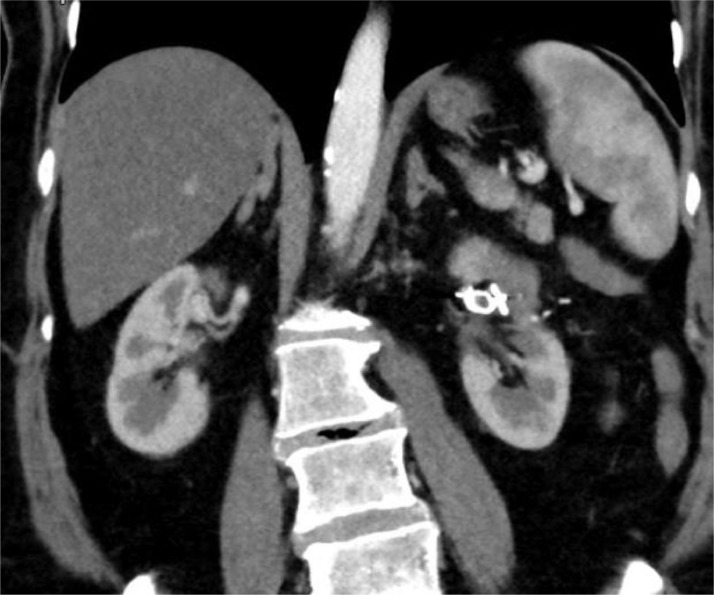
Postembolization CT arterial phase shows absence of the fistula and presence of endovascular coil

A 65-year-old woman presented to our department for an incidental diagnosis of a 35 mm totally endophytic left renal mass. Patient had no significant comorbidity serum creatinine was 0.8mg/dl and the estimated glomerular filtration rate (eGFR) was 80 mL/min. A robot-assisted PN with off-clamp technique was performed in order to obtain maximum preservation of healthy renal tissue and, consequently, of renal function. Renorrhaphy was performed with a 2 layer sliding clip technique using an uninterrupted suture with Monocryl 3-0 on the medullary layer and interrupted suture with 2-0 on the cortical layer with the use of Hem-o-Lok to fix the sutures. Total operative time was 200 minutes and no intraoperative complications were reported. The patient was discharged on postoperative day 5 with a serum creatinine of 0.9 mg/dL and an eGFR of 75 mL/min. The specimen analysis showed a clear cell renal cell carcinoma, Fuhrman grade 2, stage pT1a. The patient underwent regular follow-up evaluation according to European Association of Urology guidelines. After 1 year, CT scan showed a left AVF on the left hilar renal artery (Fig. 1). The patient was asymptomatic and her hemodynamic status was stable. Selective angiography of the left renal artery via a 6-Fr guiding catheter confirmed the presence of an AVF on the distal part of a segmental hilar branch of the artery (Fig. 2). A 12 mm Amplatzer vascular plug was loaded and delivered into the proximal dilated portion of the fistula. Following an uneventful overnight observation, the patient was discharged home. After 6 months, the follow -up is still negative and serum creatinine is 1.1 mg/dL with an eGFR of 71 mL/min (Fig. 3).

## Discussion

Acquired AVF usually occur post‐traumatically or iatrogenically including secondary to vessel puncture and surgery around vessels. First AVF following PN was reported in 1984 in a pregnant woman [2]. Etiology of AVF remains unclear and our ability to evaluate risk factors for this lesion is limited by their low incidence. However, when both an artery and a nearby vein are injured, a blood crossing from a higher-pressure system directly into the adjacent vein can occur.

Clinical manifestations can include hematuria, acute flank pain and anemia. Timing of these complications is generally limited to perioperative period, as reported in a large retrospective cohort including a total of 998 patients undergoing MIPN, where the mean interval presentation time of AVF was 14.5 days [3]. Although, late AVF presentation occurred after months from surgery have been reported in literature, highlighting the need for attentiveness to this diagnosis even several months postoperatively.

Currently, CT scan and selective angiography (SA) can provide images of the anatomy of the arteriovenous communication, typically with early contrast filling in the vein during the arterial phase. Nowadays, selective artery embolization (SAE) has become the first-line therapy for iatrogenic vascular lesions after urologic surgery. Distal renal artery branch lesions can be treated by transcatheter embolization, using a variety of embolic agents such as autologous blood clot, detachable balloons, coils, particles, and Nbutyl-2-cyanoacrylate. In our case, coils were preferred to allow a superselective embolization of the artery, in order to guarantee preservation of renal function after the treatment, as confirmed by the serum creatinine levels before and after embolization.

Several previous studies have proposed the correlation between iatrogenic vascular lesions, such as pseudoaneurysms and AVF, and preoperative nephrometry scores after nephron-sparing surgery. Notably, the most widely used R.E.N.A.L. nephrometry score system has brought to controversial results. Specifically, Omae et al. reported that the nearness of tumor deepest portion to the collecting system or sinus (N component in the R.E.N.A.L. score) is a statistically significant predictor of vascular injuries (O.R.: 1.84; 95% CI: 1.06–3.28; *P* = 0.03) [4].

Interestingly, Albani et al. reported an increased risk of iatrogenic vascular lesions such as pseudoaneurysms and AVF in minimally invasive PN compared with open PN (1.7% vs 0.4%) [5]. Reasons for this difference may include the presence of laparoscopic procedures in the MIPN cohort. With this technique, suturing can be less precise and more rapid because of concern for warm ischemia. Robotic surgery can mitigate these disadvantages with improved magnification and dexterity. Another suggestion can be that there is a decreased tightness of bolster stitches with the laparoscopic and/or robotic technique compared with the open technique. However, we believe that the sliding-clip renorrhaphy technique enables tight compression of the defect similar to that with open surgery.

## Conclusion

AVF of renal artery branches are rare complications of open and MIPN. Presentation is typically delayed from the date of the surgery, which makes it important for clinicians to consider this potentially life-threatening complication. Selective angioembolization appears to be an effective and minimally invasive management tool for these lesions, with no deleterious effect on renal function.

## Patient consent

All procedures followed were in accordance with the ethical standards of the responsible committee on human experimentation and with the Helsinki declaration of 1975, and its late amendments. Additional informed consent for publication was obtained from the patient.
